# Artificial intelligence: more human with human

**DOI:** 10.1186/s13040-017-0157-1

**Published:** 2017-12-01

**Authors:** Moshe Sipper, Jason H. Moore

**Affiliations:** 1 Institute for Biomedical Informatics, University of Pennsylvania, Philadelphia, 19104-6021 PA USA; 2 Department of Computer Science, Ben-Gurion University, Beer Sheva, 8410501 Israel

## Editorial

A layperson of our acquaintance recently brought her son to a clinic following a running injury to the foot. An X-ray was duly performed, after which not one but two medical professionals unequivocally stated that nothing seemed to be amiss. “What’s this, then?” the layperson pointed out to the experts. “Oh,” replied the latter, “that looks like a hairline fracture.”

“Deep learning,” one is tempted to vociferate at this point, “would surely *not* have averred such a crass misdiagnosis!” Indeed, in a recent paper, digital radiology pioneers Michael Recht and R. Nick Bryan wrote that, “machine learning/artificial intelligence, will be a boon to radiologists by increasing their value, efficiency, accuracy, and personal satisfaction” [1].

But why stop at radiology? It is not hard to imagine an advanced med-couch, where the patient relaxes comfortably while Artificial Intelligence (AI) algorithms diagnose the irksome hairline fracture, whereupon a 3D printer produces a cast, which a robotic arm proceeds to place posthaste. And all this done sans humans.

We argue herein that one should not be too quick to oust humans from the AI loop. First, while humans may be fallible—so is AI. Sometimes, even changing a single pixel might cause a deep neural network to misclassify an image of an automobile as a dog [2]. AI pioneer Geoffrey Hinton recently stated that AI research needs to start over [3]. According to Hinton, supervised learning requires mountains of labeled data, which is not how the brain works. He suggested that AI needs to move to unsupervised learning. We humans are quite good at learning even when labels are in short supply.

The biomedical field in particular must be wary of human-absent systems. It is one thing when an AI system errs by placing an inappropriate ad on your social medium feed or by proposing a route home that is ten minutes longer than the shortest one, quite another when such a system errs by misdiagnosing cancer. Mistakes in medicine are often costly, both financially and physically.

As argued by Ronald and Sipper (quite some time before the current spate of AI research): Intelligence is not enough [4, 5]. “In the long run,” they wrote, “we contend that the question of... intelligence will cede its place to more burning issues, arising from the use of these [AI systems]...” Specifically, they identified four issues, which are pertinent to our current discussion:

1. Trust: Can we come to trust an AI system to a degree commensurate with the trust we place in a human being? If a machine tells you that heart surgery is in order, would you trust it to the same degree you trust your cardiologist?
2. Sociality: Humans coevolve alongside their technology (witness our adapting to the continuous use of smartphones). Given our innate, evolved social nature, what role will AI systems play in the social whirlpool?
3. Identity: How does one imbue an AI system with a recognizable, temporally stable mind? Your doctor looks the same when you come back for re-evaluation after one week. But a machine may assume different forms at different times. What if your virtual doctor looked human one day, only to become “a grinning chimpanzee twirling a stethoscope” [5] the next day? This is not mere fanciful, gedankenexperiment thinking, but speaks to the very core of our evolved, biological ability to interact with other humans. Interacting with machines is an altogether different matter.
4. Responsibility: What does it mean to hold an AI system accountable for its actions? Where medical AIs are concerned, issues of liability, insurance, legality, and so forth, rear their heads very quickly.

The AI hype (some would say overhype), disseminated on a daily basis, often seems to point to the imminent dethroning of humans by their AI offspring.

Let us end emphatically: Report of our death is an exaggeration.

## Abbreviations

AI: Artificial intelligence
